# Yield components affected by rice black-streaked dwarf virus disease in rice cultivars with different resistance levels

**DOI:** 10.3389/fmicb.2023.1323569

**Published:** 2023-12-14

**Authors:** Yue Sun, Dedao Jing, Jiayuan Zhang, Linlin Du, Chenyang Li, Ying Lan, Feng Lin, Tong Zhou

**Affiliations:** ^1^Jiangsu Key Laboratory for Food Quality and Safety-State Key Laboratory Cultivation Base, Institute of Plant Protection, Jiangsu Academy of Agricultural Sciences, Nanjing, Jiangsu, China; ^2^College of Food Science, Shenyang Agricultural University, Shenyang, Liaoning, China; ^3^Zhenjiang Institute of Agricultural Sciences of the Ning-Zhen Hilly District, Zhenjiang, Jiangsu, China; ^4^International Joint Center for Japonica Rice Research, Nanjing, Jiangsu, China

**Keywords:** rice, yield component, RBSDVD, resistance, breeding

## Abstract

**Introduction:**

Rice black-streaked dwarf virus disease (RBSDVD) is one of the most destructive rice viral diseases, leading to severe yield losses in rice production. However, little is known about the yield-related components associated with the disease and no resistance cultivars have been successfully used in rice breeding.

**Methods:**

Seven rice cultivars were analyzed in this study, including six commercial rice varieties and a new line Zhongjian No. 201 (ZJ201) containing the resistance gene *OsAP47*. Resistance levels of these cultivars were evaluated by artificial inoculation and yield components were collected, including panicle length (PL), spikelets per panicle (SPP), ripened grains per panicle (RGPP), as well as panicles per square meter (PPSM) and 1000-grain weight (TGW). Seed setting rate (SSR) were calculated with the data of SPP and RGPP.

**Results and discussion:**

The results showed that ZJ201 displayed the highest resistance level and most of the commercial rice cultivars exhibited susceptible to RBSDVD. Yields of all the rice cultivars were significantly declined except ZJ201 and yield losses produced by RBSDVD were mainly due to the reduction of PL, SPP, RGPP, and TGW, suggesting that developments of these traits are associated with RBSDV infection. Resistant rice cultivar could reduce yield losses by maintaining normal development of these traits. Significant correlations were identified between resistance levels and the yield components except SSR and PPSM. The results provided useful clues for understanding the mechanisms of RBSDV invasion and its effect on rice production. ZJ201 was demonstrated as a resistance material that could be used in rice breeding.

## 1 Introduction

Rice (*Oryza sativa*) is one of the most important food crops, and approximately half of the world population is wholly dependent upon it as a staple crop for nutrition and caloric intake, contributing more than one-fifth of the calories consumed by humans. Owing to the rapid population growth, the current crop yield stability requires an attenuation of the reduction in yield and quality losses caused by environmental stress, owing to shifts in pathogens and other factors (Bailey-Serres et al., 2019; Buresh et al., 2021). Plant pathogens, including viruses, bacteria, fungi, and so forth, cause a total yield loss of ~30% in staple food crops worldwide (Wang P. et al., 2022).

Rice black-streaked dwarf virus (RBSDV) belongs to the genus *Fijivirus* in the family *Spinareoviridae* and was first reported in Japan in the early 1950s. It is transmitted by small brown planthoppers (SBPH, *Laodelphax striatellus* Fallén) in a persistent, circulative, and propagative manner. RBSDV naturally infects various *Poaceae* crops, including rice, maize, wheat, barley, and *Alopecurus aequalis* Sobal (Hibino, 1996; Zhang et al., 2001). The first major outbreaks of the disease caused by RBSDV in Japan were recorded in maize from 1957 to 1961 (Hibino, 1996).

Several outbreaks of rice black-streaked dwarf virus disease (RBSDVD) across different rice cultivars were reported in Japan (1965–1967), China (1963; 1965–1967), and Korea (1975–1976) (Hibino, 1996), resulting in severe yield losses in rice production (Ruan et al., 1984; Hibino, 1996). After disappearing for some years, RBSDVD reemerged in China between 1991 and 2002, and outbreaks have been recorded in japonica rice in several provinces in China since 2008 (Wu et al., 2020). The disease incidence (DI) of RBSDVD in rice also increased in Jeonra provinces in Korea around 2005 (Lee et al., 2005) and in Saitama Prefecture in Japan after 2010 (Matsukura et al., 2019). Up to 64,640 ha of the late japonica rice crop were affected by RBSDVD with estimated losses of 120,000 tons of grain per year from 1995 to 2007 in Zhejiang province, China (Wang et al., 2009). Since 2006, RBSDVD has been widespread in Jiangsu, Zhejiang, and Shandong provinces; outbreaks and serious epidemics were recorded during 2013–2014 in China (Ren et al., 2016). Approximately 25 and 35.6% of the planted area in Kaifeng were affected by RBSDVD in 2013 and 2014, respectively, resulting in rice yield losses of up to 200,000 kg (Ren et al., 2016).

Although severe yield losses had been observed in rice after RBSDV infection, it is not clear how yield was affected by the virus invasion, and little is known about the yield components associated with the disease. RBSDV-infected rice plants have been identified, showing pronounced stunting, darkening of leaves, twisting of leaf tips, splitting of the leaf margin, and waxy white-to-black galls along the veins on the underside of leaf blades and the outer surface of sheaths and columns (Ruan et al., 1984; Hibino, 1996). However, none of these are key components for rice yield, which is largely determined by some correlated traits such as panicle number, grain number per panicle, grain weight, and so forth (Song et al., 2022). The grain number per panicle can also be attributed to the spikelet number and seed-setting rate (SSR) of the spikelets. The development processes of these traits are regulated by genes or quantitative trait loci (QTL) involved in different pathways and networks (Xing and Zhang, 2010). In addition, these traits are usually inversely related in rice (Wang B. et al., 2018). Evaluation of the effect of RBSDV on different yield components is beneficial not only for disease control but also for revealing the mechanisms of rice responses to virus invasion.

The cultivation and application of resistance rice with high yield and good quality is an ongoing trend (Wing et al., 2018). However, genetic immunity to disease often comes with an unintended reduction in growth and yield (Ning et al., 2017). Quite a few reports have focused on RBSDVD resistance in germplasm screening and revealed a low percentage of resistant materials among the temperate sources of japonica germplasm. Only several aus, indica, and tropical japonica subspecies displayed higher levels of resistance (Pan et al., 2009; Wang et al., 2010; Zhang et al., 2016). Most of these resistance materials are local germplasms with relatively low yield and quality and have not been used successfully in rice resistance breeding. Therefore, it is necessary to evaluate the effect of resistance materials or genes on rice yield and quality.

Recently, our laboratory identified an RBSDVD-resistant rice variety, W44, and isolated a resistant gene, *OsAP47* (Wang Z. et al., 2022). With these results, an RBSDVD-resistant cultivar, ZJ201, was produced (unpublished data). Together with several susceptible rice cultivars, in this study, we aimed to assess the effect of RBSDVD on rice production and evaluate the yield-related traits of rice cultivars with different levels of resistance. The results may help in understanding the mechanisms of rice responses to RBSDV invasion and the utilization of resistance QTL or genes in rice breeding.

## 2 Materials and methods

### 2.1 Plant materials

Seven rice japonica cultivars were used in this study, namely Zhongjian No. 201 (ZJ201), Huaidao No. 5 (H5), Nanjng No. 5055 (NJ5055), Nanjng No. 9108 (NJ9108), XuDao No. 3 (XD3), Wuyunjing No. 27 (WYJ27), and Suxiu No. 867 (SX867). ZJ201 is a new rice line containing the resistance gene *OsAP47*. H5 is a late-maturing cultivar showing high and stable yield with good quality. Both NJ5055 (early maturing) and NJ9108 (late maturing) are excellent flavored cultivars. XD3 is a rice cultivar with high yield and quality that is suitable for cultivation in the Jianghuai and Huaibei areas of China. WYJ27 is a conventional cultivar that is suitable for late sowing in central Jiangsu Province. SX867 is a conventional japonica rice that is mostly planted in the Huanghuai area of China. All the rice cultivars were planted in a field in Zhenjiang, Jiangsu Province, China, during the normal sowing season in 2022.

### 2.2 Artificial inoculation of RBSDV

The seven rice cultivars were inoculated with RBSDV collected from a field in Jiangsu Province, China, and tested by polymerase chain reaction (PCR) with specific primers. The second instar SBPH nymphs were reared on the RBSDV-positive rice plants for 5 days to acquire the virus and were maintained on Wuyujing No. 3 rice seedlings, which are suitable for SBPH development. After 8 days, when the virus had circulated through the insect, the viruliferous SBPHs were detected by the dot-enzyme-linked immunosorbent assay (dot-ELISA).

At least 100 seedlings of each cultivar were grown in a room with humidity ranging from 35 to 45% and a temperature between 25° and 30°. At the 1.5-leaf stage, each seedling was fed with three viruliferous (RBSDV) or virus-free (mock) SBPHs for 3 days, as described by Li et al. (2022). Then, all insects on the plants were removed, and seedlings were cultured in the field for symptom development without pesticide or antiviral spraying. Three replicates were performed for each line. The disease incidence (the number of RBSDV-infected plants/the total number of plants counted × 100%) was recorded at 30 days post-inoculation (dpi) and repeated 7 days later. Individuals with the typical symptoms of RBSDVD were considered RBSDV-infected plants.

### 2.3 Western blot assay

Five seedlings were harvested, and mixed tissues were ground in liquid nitrogen and then extracted using protein extraction buffer (Sigma-Aldrich, St Louis, MO, USA) supplemented with protease inhibitors (phenylmethylsulfonyl fluorid (PMSF); the final concentration was 1 mM). After a 15-min centrifugation at 18,000 RCF at 4°C, the supernatant was collected from each sample and boiled for 8 min. The sample protein was separated in 10% sodium dodecyl sulfate–polyacrylamide gel electrophoresis (SDS-PAGE) through electrophoresis and then transferred onto polyvinylidene fluoride membranes. The antibody against RBSDV-CP was provided by Professor Jianxiang Wu from Zhejiang University, Zhejiang, China.

### 2.4 Yield testing

After inoculation, all the seedlings were transplanted into the experimental field in Zhenjiang, Jiangsu Province, China, during the regular planting season. Each plot consisted of one row of length of 3 m, with each row spaced 25 cm apart and 20 seedlings per row. All the plants were subjected to normal field management without antiviral spraying. At maturity, agronomic traits were collected in each plot. Plant height (PH) was measured as the average distance (cm) from ground level to the panicle tip of the tallest tiller for 10 plants. Panicle length (PL) was determined as the average length (cm) of 30 randomly selected panicles. Spikelets per panicle (SPP) represented the number of filled and empty spikelets, and ripened grains per panicle (RGPP) indicated the number of filled grains per panicle. Both SPP and RGPP were measured using 30 randomly selected panicles. Panicles per square meter (PPSM) were defined as the number of PPSM, and three replicates were counted. In each square meter, the 1,000-grain weight (TGW) was determined by measuring the weight (g) of a sample of randomly selected 1,000 grains, with three replicates per plot. SSR was calculated using the data of SPP and RGPP. Actual yields (AY) were determined by weighing all the grains of the plants in 1 sq.m, with three replicates for each plot. Theoretical yields (TY) were calculated with the following equation:


$$\begin{array}{l} \text{TY}\left(\text{kg}/\text{ha}\right)=\text{PPSM}\times 10^{4}\times \text{SPP}\times \text{SSR}\times \text{TGW}\times \;10^{-3} \end{array}$$

### 2.5 Statistical analysis

Each experiment comprised three biological replicates, and data were presented as the mean with standard deviations (SD). Differences were analyzed using a one-way analysis of variance (ANOVA). Statistical analyses were carried out using SPSS 23.0 (Armonk, USA). Different letters indicate a significant difference (with a *P*-value < 0.05).

## 3 Results

### 3.1 Different resistance levels to RBSDVD

**Figure 1:**
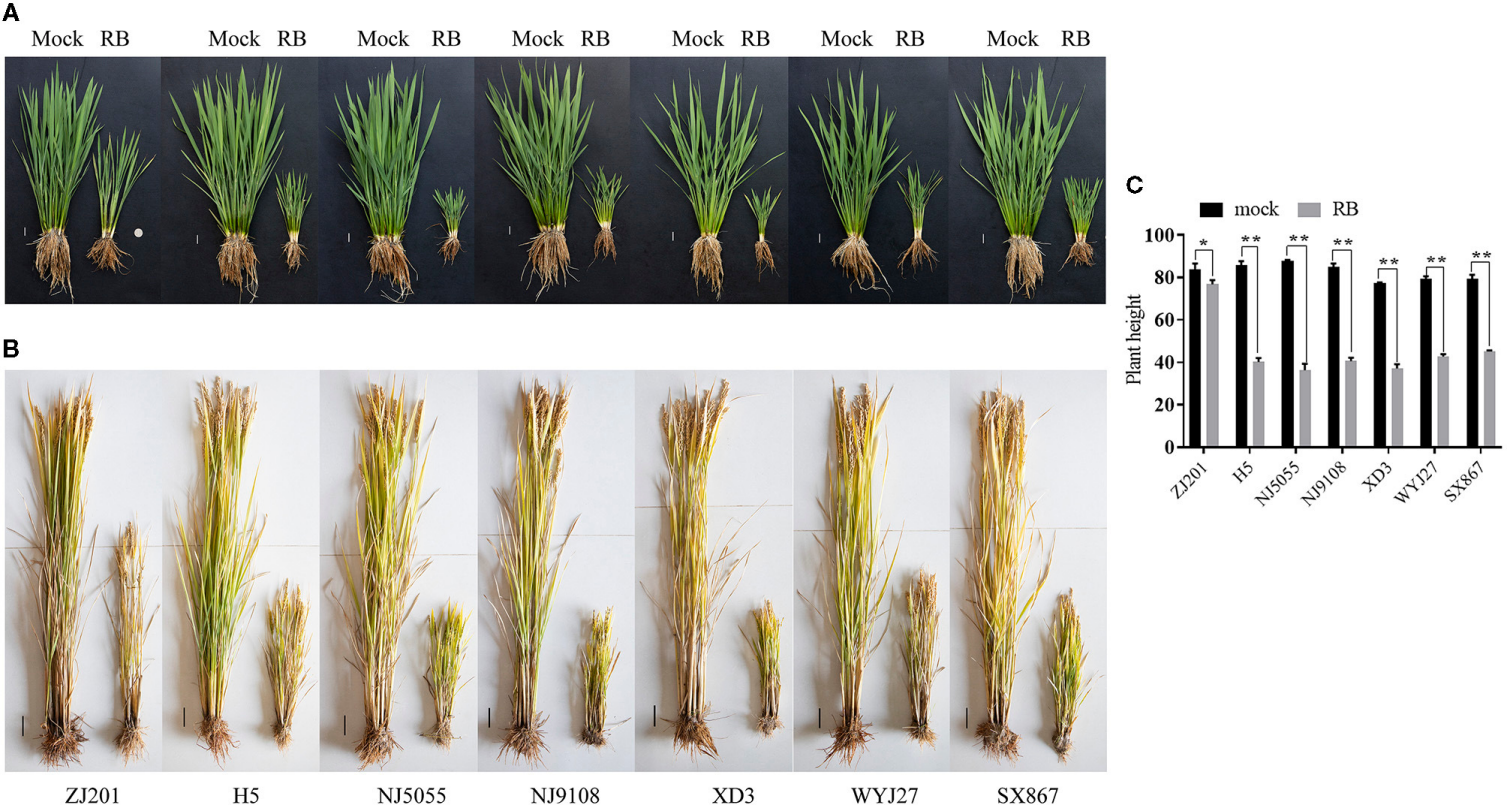
Typical symptoms of the seven rice cultivars after RBSDV infection. Phenotypes of ZJ201, H5, NJ5055, NJ9108, XD3, WYJ27, and SX867 at the jointing stage (**A**, Scale bar = 2 cm) and the maturity stage (**B**, Scale bar = 5 cm) compared with mock plants. **(C)** The plant heights of all the seven rice cultivars were significantly reduced compared with mock plants at the maturity stage. *P-value < 0.05, **P-value < 0.01.

After RBSDV infection, the rice cultivars showed typical symptoms, and the plant heights were severely stunted at the early developmental stage (Figure 1A). Plant heights of these cultivars were recorded at maturity and were ~80 cm without RBSDV infection (Figures 1B, C). The average PH of all the infected cultivars was significantly reduced compared to mock plants (Figures 1A–C). The minimum reduction was identified in ZJ201, which decreased from 83.9 to 76.4 cm. Although the PH reduction of ZJ201 reached a significant level of *P* < 0.05, the reduction rate was only 8.4%. The PH reductions of the other cultivars were significant at *P* < 0.01, with reduction rates ranging from 43.0 to 58.5%. The maximum reduction was detected in NJ5055, which decreased from 87.7 to 36.4 cm, with a reduction rate of 58.5% (Figures 1B, C).

**Figure 2:**
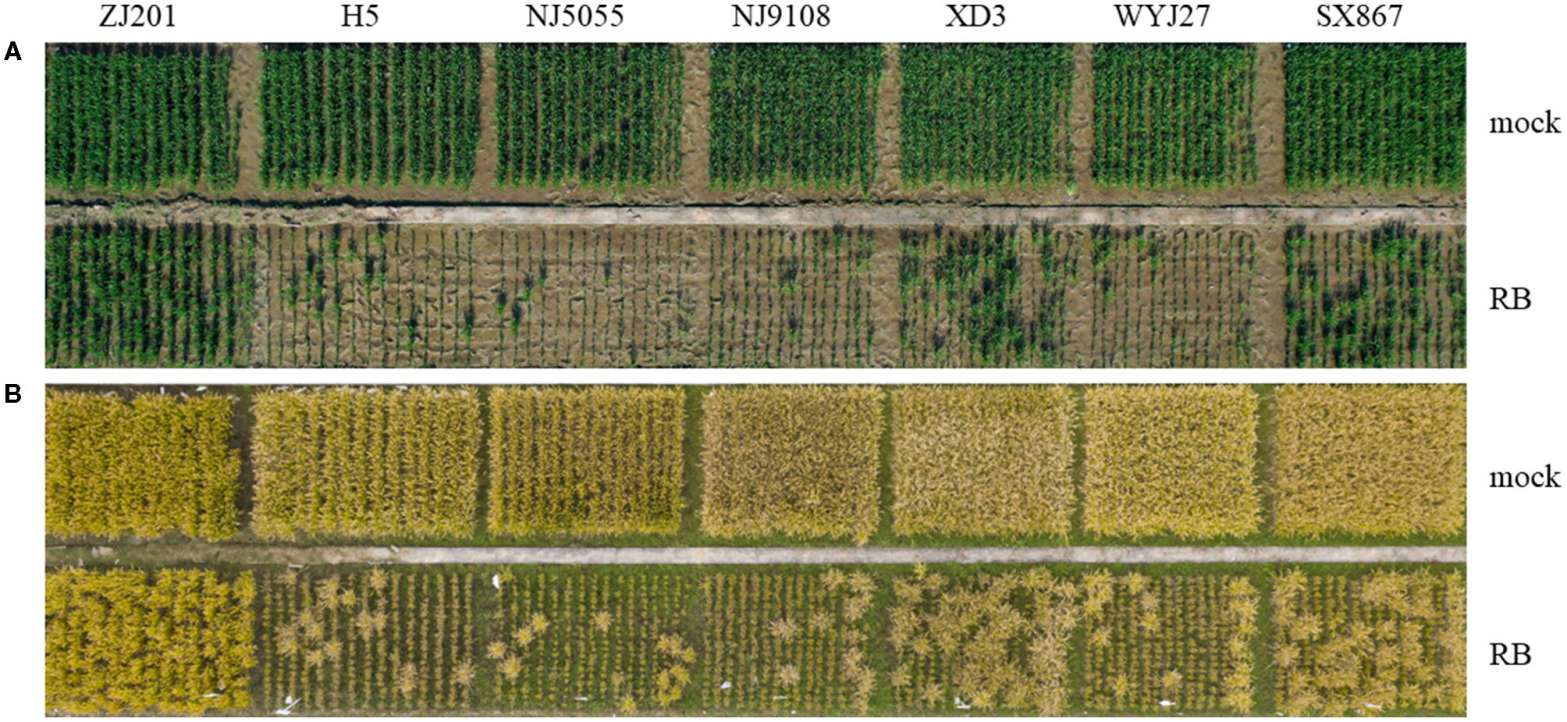
Performance of the seven rice cultivars after artificial inoculation compared with mock plants at the jointing stage **(A)** and the maturity stage **(B)**.

**Figure 3:**
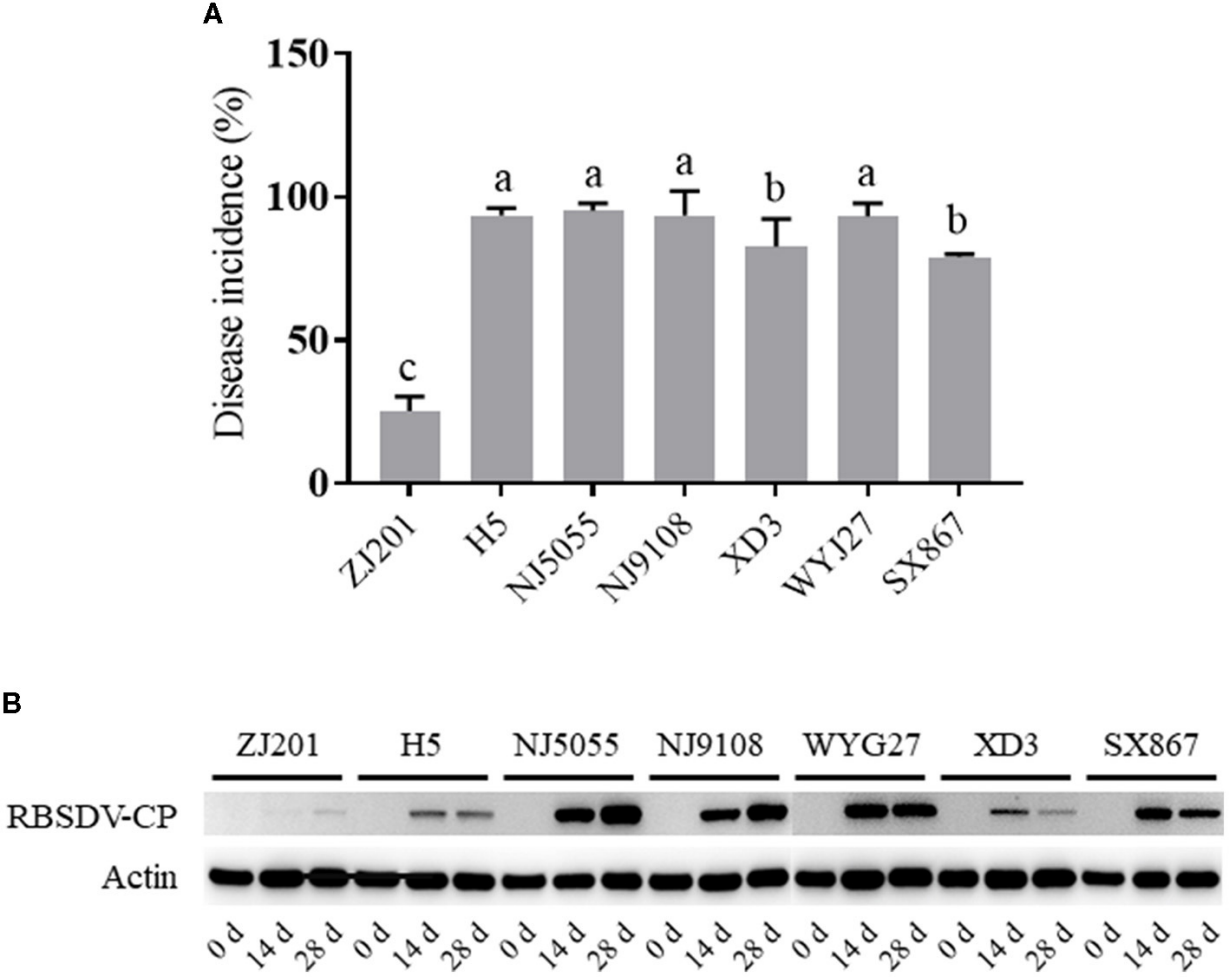
Different resistance levels of the seven rice cultivars to rice black-streaked dwarf virus disease. **(A)** Disease incidence (DI) of ZJ201, H5, NJ5055, NJ9108, XD3, WYJ27, and SX867 at 30 days post-inoculation (dpi). **(B)** Expression level of RBSDV P10 in different samples measured through Western blot assay at 0, 14, and 28 dpi. The expression of rice Actin was used as an internal control.

The infected plants with typical symptoms were recorded to calculate DIs. The rice cultivars showed different levels of resistance to RBSDVD (Figures 2A, B; Supplementary Video), with DI ranging from 25.3 to 95.3% (Figure 3A). ZJ201 displayed the highest level of resistance with DI at 25.3%, which was significantly lower than that of the other cultivars. NJ5055 exhibited the most susceptible phenotype, with DI at 95.3%. H5, NJ9108, and WYJ27 showed the same levels of susceptibility, with DI values of 93.7, 93.7, and 93.3%, respectively. The other two cultivars, SX867 and XD3, were less susceptible to RBSDVD, but the DI values were still remarkably higher than that of ZJ201 (Figure 3A).

In addition, the RBSDV protein was detected with a specific antibody against the P10 epitope, revealing the accumulation of RBSDV in these cultivars at 14 and 28 dpi (Figure 3B). The varying amounts of RBSDV accumulation suggested that resistance levels to RBSDVD were significantly different in these rice cultivars.

### 3.2 Different yield losses produced by RBSDVD

**Figure 4:**
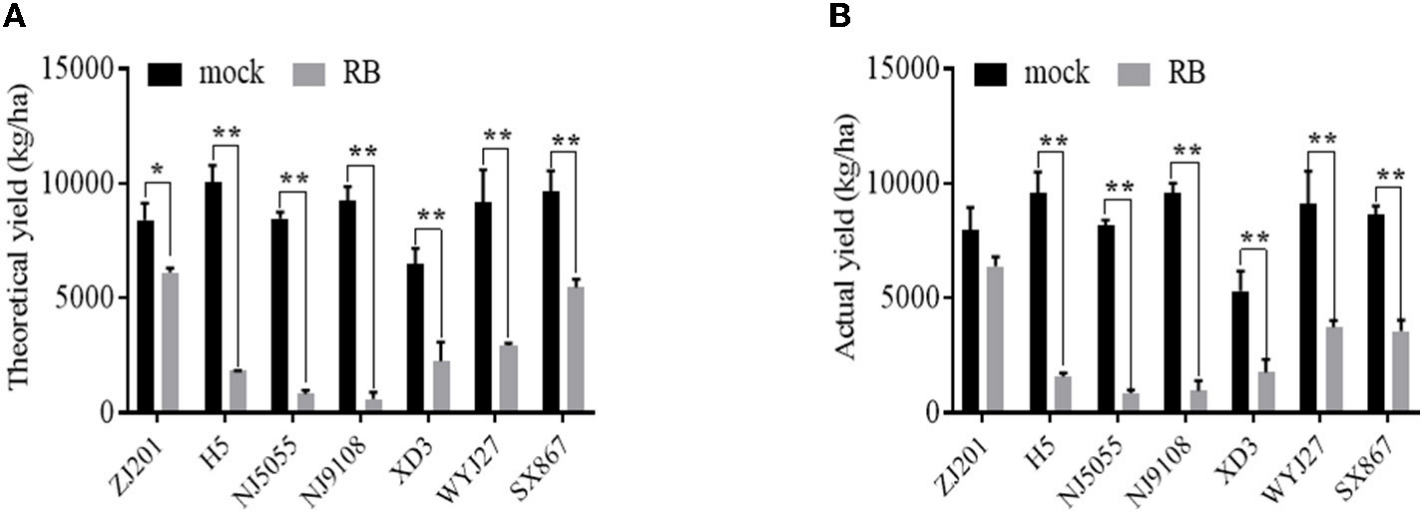
Theoretical **(A)** and actual **(B)** yield of the seven rice cultivars with (RB) or without (mock) RBSDV inoculation. *P-value < 0.05, **P-value < 0.01.

The TY and AY of mock plants ranged from 6,510.4 and 5320.3 kg/ha (XD3) to 10,063.9 and 9,607.0 kg/ha (H5). After RBSDV infection, both TY and AY significantly declined for all rice cultivars except ZJ201, reducing up to 813.6 kg/ha (NJ5055) and 850.3 kg/ha (NJ5055), respectively (Figures 4A, B). The most resistant cultivar, ZJ201, exhibited the minimum yield reduction rate in both TY (22.6%) and AY (19.9%). Although a remarkable TY reduction was detected in ZJ201 (*P* < 0.05), the AY difference did not reach a significant level.

Dramatic yield losses (*P* < 0.01) were identified in all the other rice cultivars, with the reduction rate ranging from 43.2 to 90.4% for TY and from 58.9 to 90.1% for AY. The less susceptible cultivar SX867 displayed a lower reduction rate than the other five cultivars, both in TY and AY. The reduction rate of XD3 was less than that of H5, NJ5055, and NJ9108 in both TY and AY. The susceptible cultivars NJ5055 and NJ9108 showed the most severe losses, with about a 90% reduction for both TY and AY (Figures 4A, B).

### 3.3 Yield components associated with yield losses with RBSDVD

**Figure 5:**
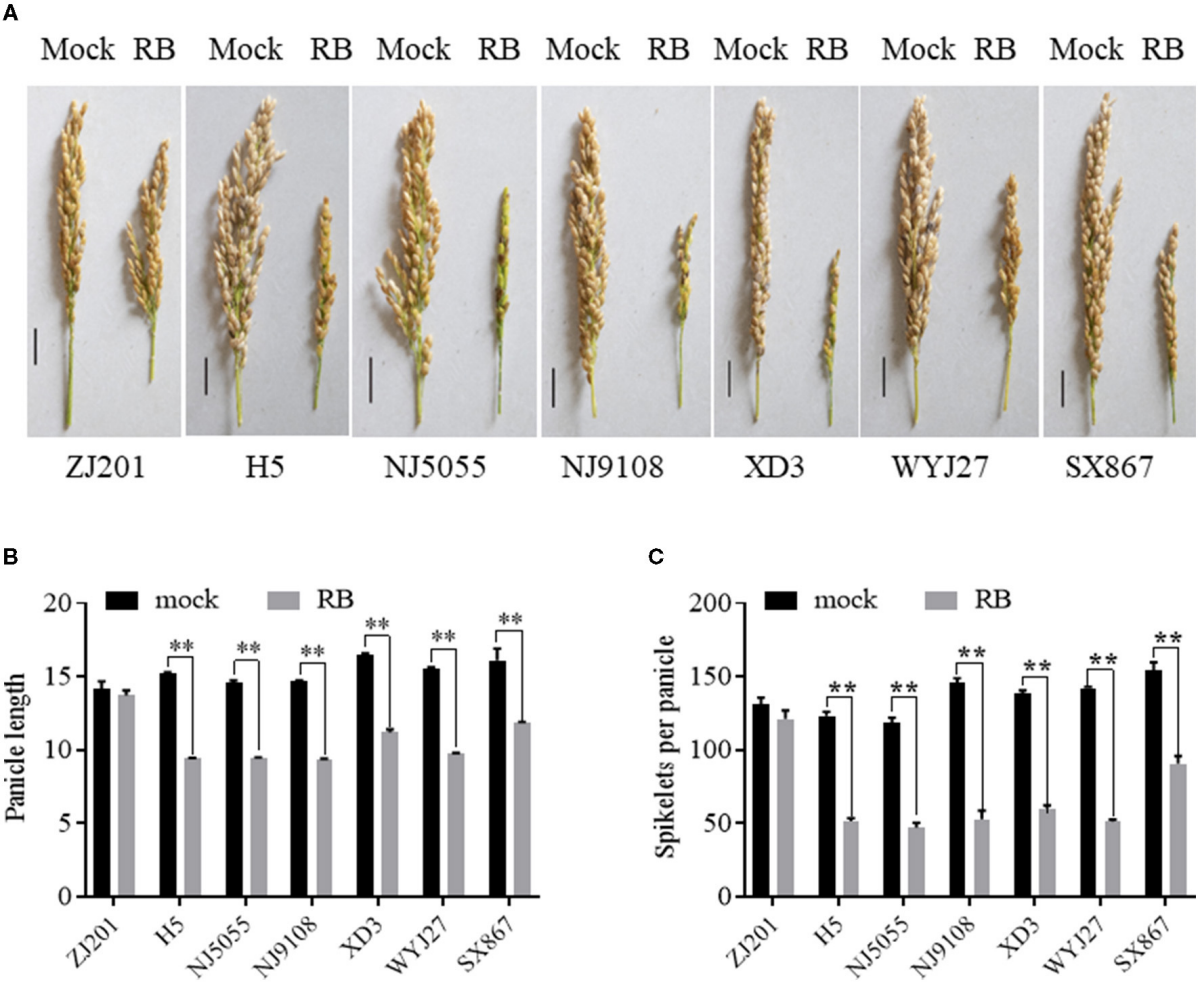
Effect of RBSDVD on panicle length and spikelets per panicle of the seven rice cultivars. **(A)** Phenotype of the panicles of inoculated and mock plants. Scale bar = 2 cm. Comparison of panicle lengths **(B)** and spikelets per panicle **(C)** of ZJ201, H5, NJ5055, NJ9108, XD3, WYJ27, and SX867 between inoculated and mock plants. **P-value < 0.01.

Rice yield is largely determined by several component traits, including panicle number, spikelet number per panicle, PL, grain weight, and so forth. PL significantly decreased after RBSDV infection for all the rice cultivars except ZJ201, with a reduction rate ranging from 26.1 to 38.1% (Figures 5A, B). No significant changes were detected between the PL data of mock and infected plants in ZJ201. The reduction rates of XD3 and SX867, with lower DI values, were 32.3 and 26.1%, respectively, less than those of the other four cultivars (35.1–38.1%). H5 displayed the maximum PL reduction from 15.3 to 9.4 cm (Figures 5A, B).

After RBSDV infection, SPPs significantly decreased for all rice cultivars except ZJ201 (Figures 5A, C). The difference between mock and RBSDV-inoculated plants did not reach a significant level in ZJ201. In the other cultivars, the reduction rate of SPP ranged from 41.2% (SX867) to 63.8% (WYJ27), all significant at *P* < 0.01 (Figure 5C). The reduction rates of XD3 and SX867 were 56.6 and 41.2%, respectively, less than those of the other four rice cultivars (58.2–63.8%). The maximum reduction was detected in WYJ27, with the SPP reduction from 142.2 to 51.5 (Figure 5C).

**Figure 6:**
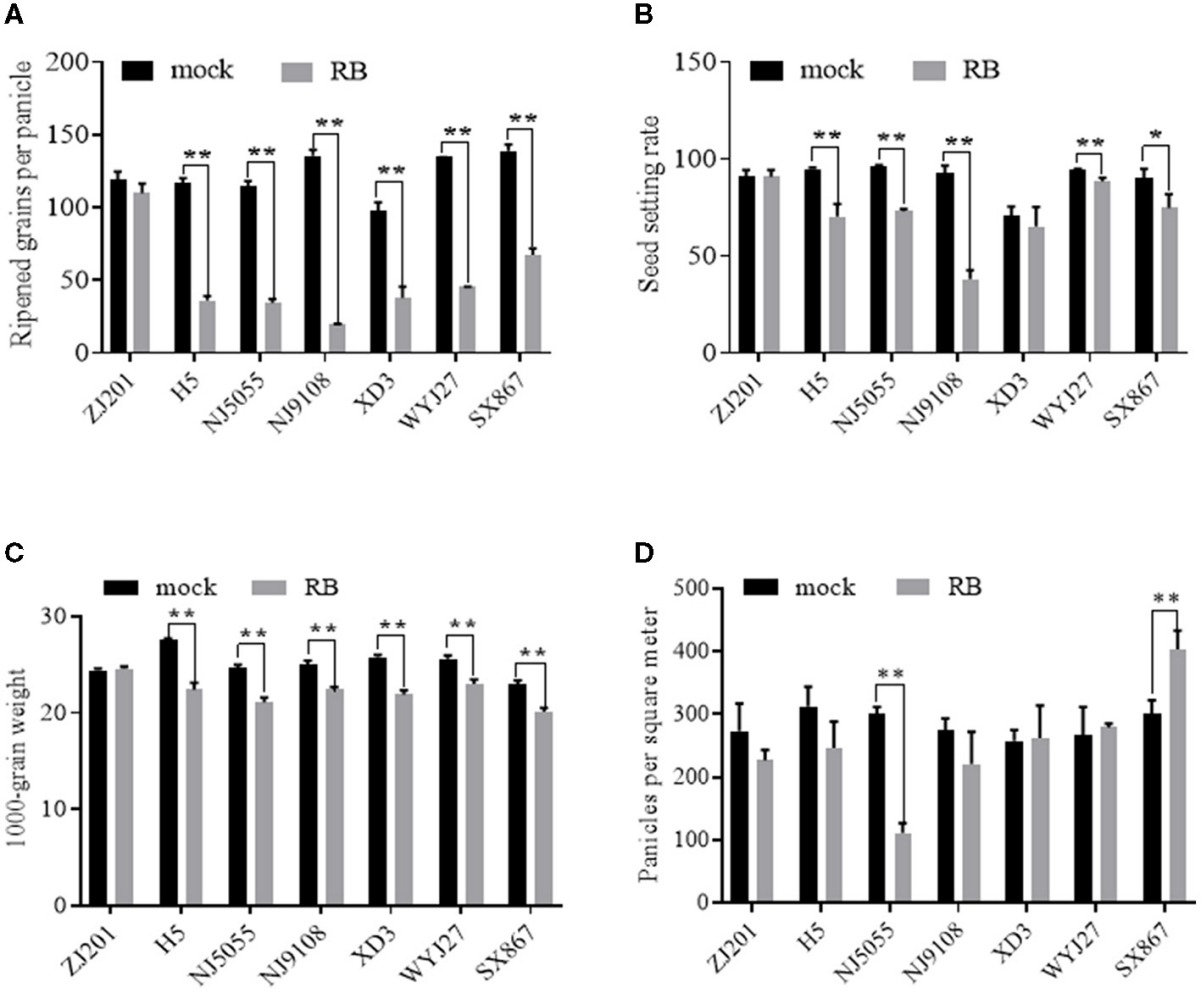
Different effects of RBSDVD on yield-contributing components: **(A)** ripened grains per panicle, **(B)** seed-setting rate, **(C)** 1,000-grain weight, and **(D)** panicles per square meter. *P-value < 0.05, **P-value < 0.01.

With the recorded data of RGPP shown in Figure 6A, SSR was calculated using the data of SPP and RGPP. No significant changes were detected between mock and RBSDV-infected ZJ201, both in RGPP and SSR (Figures 6A, B). In all the other six rice cultivars, remarkable reductions in RGPP (*P* < 0.01) were identified, and the RGPP reduction rates of XD3 (61.2%) and SX867 (51.4%) were less than those of the other cultivars (66.1–85.3%) (Figure 6A). In addition to ZJ201, changes in SSR did not reach a significant level in XD3, despite the fact that its SPP and RGPP changed remarkably (Figure 6B).

For another yield component trait, TGW, the rice cultivar ZJ201 exhibited the same level between mock and inoculated plants. TGW of the other rice cultivars declined significantly at *P* < 0.01, with the reduction rate ranging from 10.2% (WYJ27) to 18.6% (H5) (Figure 6C). H5 displayed the maximum TGW in the mock plants and the maximum reduction rate after RBSDV infection. WYJ27 displayed the minimum reduction rate from 25.6 to 23.0 g (Figure 6C).

Among the yield components affected by RBSDV, the average reduction rate in the seven rice cultivars of RGPP (58.6%) was much higher than that of PL (29.8%), SPP (50.2%), PPSM (11.5%), and TGW (11.5%), suggesting that RGPP contributes the most yield losses after RBSDV infection.

Unlike the traits described earlier, PPSM displayed diverse changes after RBSDV infection and significantly reduced only in NJ5055 but not in the other six cultivars (Figure 6D). The differences between RBSDV-infected and mock lines of ZJ201, H5, NJ9108, XD3, and WYJ27 did not reach a significant level, while that of SX867 increased after RBSDV infection (Figure 6D).

### 3.4 Yield component reductions associated with resistance levels

Given that the resistant cultivar ZJ201 displayed minimal yield losses, significant correlations were detected between DI and yield reduction rates for both TY and AY, with correlation coefficients of 0.879 and 0.904, respectively, all significant at *P* < 0.01 level (Table 1). This implies a relationship between resistance levels and rice yield production.

**Table 1:** Correlation between DI and the reduction rates of each agronomic traits.

	**TY**	**AY**	**PL**	**SPP**	**RGPP**	**TGW**	**PPSM**	**SSR**	**PH**	**LFL**
DI	0.879	0.904	0.987	0.977	0.962	0.851	0.117	0.526	0.969	0.931
*P*-value	0.009	0.005	< 0.001	< 0.001	0.001	0.015	0.803	0.225	< 0.001	0.002

Most of the yield components declined after RBSDV infection, and their reduction rates were significantly correlated with DI, including PL, SPP, RGPP, and TGW, with correlation coefficients ranging from 0.851 to 0.987 (Table 1). The other two yield-related traits, PPSM and SSR, did not show a remarkable correlation with DI, a correlation coefficient of 0.117 and 0.526, respectively (Table 1).

## 4 Discussion

To reduce the impact of RBSDVD in regions with epidemics, various measures were produced, which were primarily aimed at interrupting the cycle of virus transmission by the vector SBPHs. These measures included covering rice seedling nurseries with insect-proof nets or applying pesticides to control the insects (Wu et al., 2020). However, these management practices tend to waste natural resources or cause environmental pollution, thereby posing challenges. Identifying natural resistance resources and breeding resistant varieties is considered the most economical and effective approach to control this disease.

According to a 3-year evaluation, three varieties were identified with consistent DI of < 10%, and all originated from Southeast Asia (Wang et al., 2014). Four rice varieties with stable levels of RBSDV resistance were discovered in the rice diversity panel 1 cultivars (Feng et al., 2019). Recently, our laboratory identified a highly RBSDVD-resistant variety, W44, through an extensive evaluation of a diverse international rice panel and isolated a resistance gene, *OsAP47* (Wang Z. et al., 2022). To utilize these resistance germplasms in rice resistance breeding, our laboratory bred a new line, ZJ201, based on the resistant cultivar W44, and introduced *OsAP47* into a new genetic background. In this study, seven rice cultivars were evaluated, and ZJ201 displayed the highest level of resistance against RBSDVD, as indicated by DI (Figure 3).

As expected, yields of all rice cultivars declined after RBSDV infection, with losses of up to 90.1% in AY, implying significant risks associated with the use of these popular rice varieties at present. ZJ201 displayed minimal reduction, and the difference in AY did not reach a significant level. These results confirmed that breeding resistant rice cultivars could minimize the yield losses caused by RBSDVD. The significant correlations detected between DI and yield reduction suggest that DI could serve as a good indicator for RBSDVD resistance levels and yield potentials.

The interaction between viruses and host plants adversely affects host morphology and physiology, leading to a decline in production. Rice yield is a complex trait determined by various components, primarily including the number of panicles per plant, the number of grains per panicle, and grain weight (Xing and Zhang, 2010). These components were regulated by different genes or QTLs (Li et al., 2000; Hittalmani et al., 2003; Wang et al., 2011; Huang et al., 2013; Tan et al., 2013; Yano et al., 2016), implying their involvement in diverse pathways. However, little is known about which pathway is affected by RBSDV invasion. Among the yield components detected in this study, most of them were reduced, such as PL, SPP, RGPP, and TGW, compared to mock plants after RBSDV infection, suggesting that these components were significantly affected by RBSDV.

Two yield components, SSR and PPSM, did not show a reduction in all rice cultivars after RBSDV infection. SSR significantly declined at levels of *P* < 0.01 in H5, NJ5055, NJ9108, and WYJ27 and of *P* < 0.05 in SX867. However, in XD3, despite both SPP and RGPP being significantly affected by RBSDVD, the changes in SSR between mock and inoculated plants did not reach a significant level (Figure 6B). The other component, PPSM, was significantly reduced in NJ5055 but dramatically increased in SX867, and no remarkable differences were detected in ZJ201, H5, NJ9108, XD3, and WYJ27 (Figure 6D). These results suggested that the yield losses produced by RBSDVD were mainly due to the reduction in PL, SPP, RGPP, and TGW but not SSR and PPSM. Similar results were observed in wheat, where the yield component most severely affected by barley yellow dwarf virus infection was the number of kernels per spike, followed by kernel weight, while the tiller number was generally not altered by the infection (Hoffman and Kolb, 1998). These results provided important clues for understanding the mechanisms of RBSDV invasion and its effects on rice after infection.

Active immune response in crops fighting pathogens may result in yield penalties (Wang J. et al., 2018). A genome-wide association study was conducted to assess the association between rice blast disease resistance and yield-related components; the analysis results showed that shorter plants were significantly associated with resistance to blast. Additionally, rice genomes with resistance genes were linked to lighter seed weights, while susceptible alleles were associated with heavier seed weights (Wang et al., 2015). In this study, we evaluated the rice line ZJ201, which carries the RBSDVD resistance gene *OsAP47*. The results indicated that ZJ201 did not have a notable negative effect on the main yield-contributing characters, for instance, SPP, RGPP, SSR, TGW, and PPSM, thereby producing a higher yield. These observations could facilitate the application of *OsAP47* to avoid yield losses caused by RBSDVD. In the future, quality traits will be evaluated for different rice cultivars after RBSDV infection, and ZJ201 will be assessed for its utilization in rice resistance breeding.

## 5 Conclusion

Rice yield could be significantly reduced by RBSDV infection, mainly by affecting PL, SPP, RGPP, and TGW. Among these, RGPP contributed the most to yield losses. Resistant rice lines could avoid yield losses by maintaining the normal development of these traits. ZJ201 could be promoted for planting in areas severely affected by RBSDVD or used as a resistance intermediate material in rice breeding.

## Data availability statement

The original contributions presented in the study are included in the article/Supplementary material, further inquiries can be directed to the corresponding authors.

## Author contributions

YS: Data curation, Writing – original draft, Investigation. DJ: Resources, Writing – original draft. JZ: Data curation, Writing – original draft. LD: Investigation, Writing – original draft. CL: Methodology, Writing – original draft. YL: Resources, Writing – original draft. FL: Conceptualization, Data curation, Investigation, Project administration, Supervision, Writing – original draft, Writing – review & editing. TZ: Conceptualization, Funding acquisition, Project administration, Supervision, Writing – review & editing.

## Supplementary material

Supplementary Video Performance of the seven rice cultivars at the jointing stage. Top: mock plants, bottom: artificially inoculated plants. From left to right: ZJ201, H5, NJ5055, NJ9108, XD3, WYJ27, and SX867.
